# Inappropriate Antimicrobial Dosing in Regard to Renal Function in a Tertiary Hospital in Greece—A Single-Center Point Prevalence Study

**DOI:** 10.3390/medicina62040743

**Published:** 2026-04-13

**Authors:** Petros Ioannou, Andria Papazachariou, Stamatis Karakonstantis, Diamantis Kofteridis

**Affiliations:** 1 School of Medicine, University of Crete, 71003 Heraklion, Greece; 2 Internal Medicine Department, University Hospital of Heraklion, 71110 Heraklion, Greece

**Keywords:** antimicrobial, antibiotic, kidney function, renal function, antimicrobial stewardship, point-prevalence study

## Abstract

*Background and Objectives*: Appropriate antimicrobial dosing according to kidney function is essential to ensure therapeutic efficacy while minimizing toxicity and antimicrobial resistance. Despite established dosing guidelines and electronic prescribing systems, errors in renal dose adjustment of antimicrobials, particularly in the setting of acute kidney injury, remain common among hospitalized patients. *Materials and Methods*: A point-prevalence study was conducted on 31 October 2024 at a tertiary-care hospital in Greece to evaluate the appropriateness of antimicrobial dosing in relation to renal function. Patient characteristics, renal parameters, and antimicrobial prescriptions were extracted from electronic medical records. Glomerular filtration rate (GFR) was estimated using the MDRD formula. Comparative analyses were performed between correctly and incorrectly dosed cases, and between overdosing and underdosing episodes. *Results*: A total of 235 hospitalized patients were evaluated (mean age 64.8 ± 18.6 years; 43.4% female). Overall, 15.7% (37/235) received at least one antimicrobial dose inappropriate for their renal function. Among 37 patients where dosing errors were identified, overdosing was noted in 23 (62.2%), underdosing in 16 (43.2%), adding up to 39 prescriptions, while in 2 patients (5.4%), both mistakes were noted in different prescribed antimicrobials. Drug-specific error rates varied considerably: ceftazidime and cefuroxime showed the highest rates of inappropriate dosing (40% each), followed by colistin (33.3%) and acyclovir (33.3%). Piperacillin/tazobactam, the most frequently prescribed agent (*n* = 50), had a 14% error rate, mainly due to underdosing (10%). Patients with dosing errors were significantly older (71.5 vs. 64.1 years, *p* = 0.0220) and had worse renal function, including higher serum creatinine (1.68 vs. 1.19 mg/dL, *p* = 0.0174), lower GFR (58.5 vs. 75.9 mL/min/1.73 m^2^, *p* = 0.0009), and more frequent dialysis (13.5% vs. 4.3%, *p* = 0.0422). They also received a higher median number of antimicrobials (2 vs. 1, *p* = 0.0185). *Conclusions*: Inappropriate antimicrobial dosing based on kidney function remains common in hospitalized patients, particularly among older individuals and those with impaired renal function or polypharmacy. Targeted antimicrobial stewardship strategies focusing on renal dose adjustment and agents that are more frequently dosed inappropriately, such as colistin, acyclovir, cefuroxime, and ceftazidime, as well as agents that are frequently prescribed despite a relatively lower rate of inappropriate dose, such as piperacillin/tazobactam, are needed to enhance prescribing safety and optimize therapeutic outcomes.

## 1. Introduction

Antimicrobials are among the most frequently prescribed medications in hospitalized patients, with studies showing that at least one in three hospitalized patients at any given time may be receiving antimicrobials [1,2,3,4]. Optimizing antimicrobial dosing is a cornerstone of antimicrobial stewardship, as inappropriate dosing may lead to therapeutic failure, drug toxicity, emergence of resistance, and increased healthcare costs [5]. Among the factors influencing antimicrobial dose selection, kidney function plays a particularly critical role, since many commonly used antimicrobials are primarily eliminated through renal excretion [6].

Antimicrobial dosing optimization is particularly important in patients with altered pharmacokinetics, such as those with impaired renal function [7,8]. A large proportion of commonly used antimicrobials—including β-lactams, aminoglycosides, glycopeptides, fluoroquinolones, and antiviral agents—are primarily eliminated through renal excretion [9]. Consequently, reductions in glomerular filtration rate (GFR) may lead to significant drug accumulation if doses are not appropriately adjusted. This accumulation increases the risk of dose-dependent toxicity, which may manifest as nephrotoxicity, neurotoxicity, or other adverse drug reactions depending on the antimicrobial agent involved. For example, accumulation of β-lactams has been associated with encephalopathy and seizures. At the same time, drugs such as acyclovir and colistin have well-recognized nephrotoxic potential in the setting of impaired renal clearance [10,11,12,13,14,15,16].

In addition to chronic kidney disease (CKD), acute kidney injury (AKI) is frequently encountered in hospitalized patients and further complicates antimicrobial dosing decisions. Renal function in these patients may fluctuate rapidly due to infection severity, hemodynamic instability, exposure to nephrotoxic agents, or critical illness-related physiological changes. As a result, antimicrobial doses that were appropriate at the initiation of therapy may become inappropriate later during hospitalization [17,18]. This dynamic nature of renal function underscores the importance of frequent monitoring and timely dose adjustment during treatment.

Conversely, excessive dose reduction may also have detrimental consequences. Subtherapeutic antimicrobial exposure may lead to treatment failure, prolonged infection, and the selection of antimicrobial-resistant organisms [19,20]. This issue is particularly relevant for time-dependent antimicrobials such as β-lactams, where maintaining adequate drug concentrations above the minimum inhibitory concentration (MIC) is essential for optimal efficacy [21]. Inadequate dosing strategies may therefore compromise both individual patient outcomes and broader public health efforts to contain antimicrobial resistance.

Despite the availability of published dosing guidelines, institutional protocols, and computerized decision-support tools, several studies have demonstrated that inappropriate antimicrobial dosing in patients with renal impairment remains frequent in clinical practice. Reported rates vary widely across healthcare settings, ranging from approximately 10% to more than 40% of prescriptions depending on study design, patient population, and the antimicrobial agents evaluated. Factors contributing to dosing errors include prescriber unfamiliarity with dosing recommendations, complex dosing regimens, inaccurate estimation of renal function, and the absence or limited use of clinical decision support systems [22,23].

Point prevalence surveys (PPS) represent a useful methodology for evaluating antimicrobial prescribing practices at the institutional level. PPS studies allow the systematic assessment of antimicrobial utilization patterns and can identify specific targets for antimicrobial stewardship interventions. Although PPS studies have been widely used to assess antimicrobial consumption and the appropriateness of indications, relatively few have focused specifically on the appropriateness of antimicrobial dosing based on renal function [24,25].

Given the increasing prevalence of chronic kidney disease in aging populations and the high burden of infections among hospitalized patients, ensuring appropriate antimicrobial dosing in this population represents an important clinical and stewardship priority. Understanding local prescribing practices and identifying improved dosing error patterns thereby improves medication safety and therapeutic effectiveness.

The present study aimed to evaluate the appropriateness of antimicrobial dosing in relation to kidney function in a tertiary-care hospital in Greece using a point-prevalence design. Additionally, we sought to compare characteristics of patients receiving correct versus incorrect doses, distinguish between overdosing and underdosing, and assess drug-specific patterns of dosing errors.

## 2. Materials and Methods

A point-prevalence study (PPS) was conducted at the University General Hospital of Heraklion, Greece, on 31 October 2024. The University General Hospital of Heraklion is a tertiary-care academic hospital that serves as a referral center for the island of Crete and the surrounding regions. It has about 720 beds and an Infectious Diseases service that cares for patients after they are consulted by the primary physicians who care for them. Antimicrobial stewardship during the study period was performed only during the Infectious Diseases consultation, without having a hospital-wide unsolicited protocol. The hospital includes a wide range of medical and surgical specialties and provides advanced care, including intensive care services, dialysis support, and complex surgical procedures. At the time of the study, antimicrobial prescriptions were recorded through an electronic medical record system that allowed access to patient demographics, laboratory results, and medication orders.

More specifically, for the purpose of this PPS, all hospitalized patients receiving at least one systemic antimicrobial agent on the study date were evaluated. Antimicrobials included antibacterial, antifungal, and antiviral agents administered irrespective of the method of administration. Topical agents applied to the skin were not included.

Data extracted from the electronic medical records included patient demographic characteristics (age and sex), hospital ward, renal function parameters (serum creatinine and estimated GFR), dialysis status, route of antimicrobial administration, and the number and type of antimicrobials prescribed. For each antimicrobial agent, the prescribed dose and dosing interval were recorded. Glomerular filtration rate (GFR) was estimated using the MDRD formula [26,27].

The appropriateness of antimicrobial dosing was evaluated by infectious diseases specialists by comparing the prescribed regimen with established dosing recommendations adjusted for renal function, as outlined in widely used clinical references such as the Sanford guide (primary tool used for resolution of any discrepancies), institutional protocols, and published guidelines [28,29,30]. Both the administered dose and dosing interval were considered when determining appropriateness. Prescriptions were classified as correct, overdosed, or underdosed relative to the recommended dosing for the patient’s estimated renal function category. When a combination of antimicrobials was used, each antimicrobial was assessed individually.

Descriptive statistics were applied to summarize the findings. Continuous variables were compared using Student’s *t*-test for normally distributed variables, after assessing normality with the D’Agostino & Pearson omnibus normality test, and the Mann–Whitney test for non-normally distributed variables, and categorical variables were compared with chi-square tests. All tests were two-tailed, and *p*-values of 0.05 or less were considered significant. Statistics were calculated with GraphPad Prism 6.0 (GraphPad Software, Inc., San Diego, CA, USA). A multivariate logistic regression analysis of inappropriate dosing was performed, including all the parameters that were identified to be different in a statistically significant manner between appropriately and inappropriately dosed antimicrobial prescriptions. Multivariate analysis was performed using SPSS version 23.0 (IBM Corp., Armonk, NY, USA).

The study was conducted in accordance with the Declaration of Helsinki and was approved by the Institutional Review Board of the University Hospital of Heraklion (22/13-08-2024).

## 3. Results

### 3.1. Overall Patient Population and Renal Profile

The point-prevalence study included 235 patients hospitalized at the University General Hospital of Heraklion. Table 1 shows the patients’ characteristics overall and whether antimicrobial dosing was correct. The study population had a mean age of 64.8 years (standard deviation (SD): 18.6), and 43.4% (*n* = 102) were female. Clinical distribution across the hospital showed that 51.5% (*n* = 121) of patients were in medical wards, 46% (*n* = 108) in surgical wards, and 2.6% (*n* = 6) in the Intensive Care Unit (ICU).

The renal function profile of the cohort was characterized by a mean serum creatinine of 1.27 mg/dL (SD: 1.2) and a mean GFR of 73.1 mL/min/1.73 m^2^ (SD: 29.4). Additionally, 5.1% (*n* = 12) of the total patient population was undergoing dialysis at the time of the study. Regarding treatment administration, the vast majority of antimicrobials were delivered intravenously (87.8%), while 11.6% were administered orally.

### 3.2. Comparative Analysis of Dosing Appropriateness

**Figure 1:**
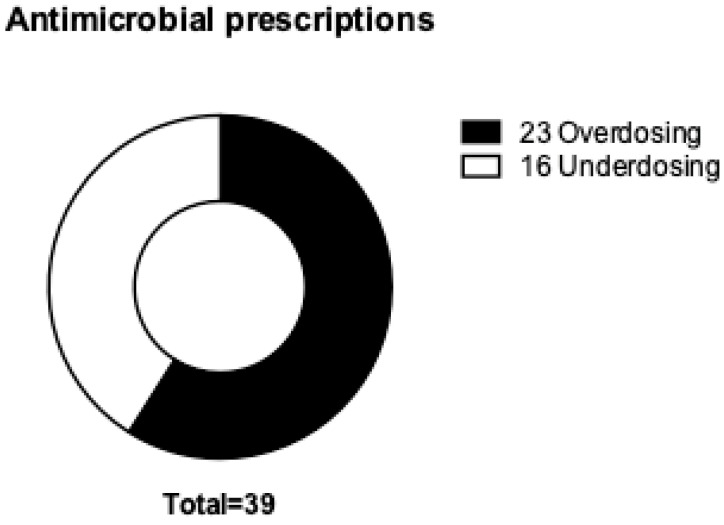
Distribution of overdosing and underdosing.

Out of the 235 patients, 37 (15.7%) were identified as receiving an incorrect antimicrobial dose based on their kidney function. Table 2 shows the patients’ characteristics in terms of whether antimicrobial dosing was over or under the indicated dose based on the patients’ kidney function. Several significant differences emerged between patients who received correctly dosed antimicrobials (*n* = 209) and those who received incorrect doses (*n* = 37). Patients receiving incorrect doses were significantly older, with a mean age of 71.5 years compared to 64.1 years in the correctly dosed group (*p* = 0.0220). Markers of kidney function were significantly worse in the group treated with incorrect doses, including higher serum creatinine (1.68 vs. 1.19 mg/dL; *p* = 0.0174) and lower GFR (58.5 vs. 75.9 mL/min/1.73 m^2^; *p* = 0.0009). A significantly higher proportion of patients on dialysis received an incorrect dose (13.5%) than those not on dialysis (4.3%; *p* = 0.0422). The median number of antibiotics prescribed was significantly higher among patients with dosing errors (median: 2; IQR: 1–3) than among those with correct dosing (median: 1; IQR: 1–2; *p* = 0.0185). No significant differences were found regarding gender (*p* = 0.1541), ward type (*p* = 1 for medical; *p* = 0.8590 for surgical), or the route of administration. Among the 39 instances (among 37 patients, 2 of whom had more than one incorrectly dosed prescription of antimicrobials) of inappropriate dosing (noted across various metrics), 23 cases were categorized as overdosing and 16 cases as underdosing, as shown in Figure 1.

Patients who were overdosed were significantly older (mean: 75.6 years) than those who were underdosed (mean: 65.8 years, *p* = 0.0369). Furthermore, overdosing was significantly more prevalent in medical wards (65.2%) compared to underdosing (25%, *p* = 0.0225). Conversely, underdosing was more frequent in surgical wards (62.5%) than overdosing (34.8%). Renal function also differed between these groups, since the overdosed group had a significantly lower mean GFR (49 mL/min/1.73 m^2^) compared to the underdosed group (74.1 mL/min/1.73 m^2^, *p* = 0.0108).

A multivariate logistic regression analysis of inappropriate dosing was performed including all the parameters that were identified to be different in a statistically significant manner between appropriately and inappropriately dosed antimicrobial prescriptions. Table 3 shows the results of the multivariate analysis. Only low GFR was an independent predictor of inappropriate dosing of antimicrobials. More specifically, for each added mL/min/1.73 m^2^, there was an odds ratio of 0.979 for inappropriate dose of antimicrobials.

### 3.3. Specific Antimicrobial Performance

The error rates for the most commonly prescribed antimicrobials provided insight into specific prescribing challenges. Table 4 shows data on the most commonly prescribed antimicrobials with incorrect dosing. Piperacillin/tazobactam was the most frequently prescribed agent (*n* = 50), with a total error rate of 14%, predominantly due to underdosing (10%) rather than overdosing (4%). Regarding cephalosporins, high rates of inappropriateness were seen with ceftazidime (40% error rate, entirely due to overdosing) and cefuroxime (40% error rate, entirely due to underdosing). Cefoxitin had an error rate of 11.4%, with underdosing (8.6%) being more common than overdosing (2.9%). Regarding other agents, colistin showed a 33.3% error rate, split equally between over- and underdosing (16.7% each). Acyclovir also had a high error rate of 33.3%, all of which were attributed to overdosing. Ciprofloxacin and meropenem had total error rates of 18.2% and 8%, respectively.

## 4. Discussion

In this single-center PPS, approximately one in six hospitalized patients receiving antimicrobials received an inappropriate dose based on kidney function. This prevalence is consistent with other studies, also showing inappropriately dosed antimicrobials with rates depending on the patient population, study design, and definitions used [23,31,32]. Despite widespread availability of dosing guidelines and electronic prescribing systems, renal dose adjustment errors remain common in routine clinical practice, both in inpatients and in ambulatory patients [33].

The prevalence of inappropriate antimicrobial dosing observed in this study (15.7%) is alarming, but broadly consistent with findings from previous studies conducted in various healthcare settings. Earlier investigations have reported error rates ranging from approximately 10% to over 40%, depending on differences in patient populations, definitions of dosing appropriateness, and antimicrobial agents evaluated [23,31,32,34]. Studies focusing specifically on patients with chronic kidney disease or acute kidney injury have often reported even higher rates of dosing inappropriateness, highlighting the challenges associated with managing antimicrobial therapy in these populations [23]. For example, analyses of hospitalized patients with renal impairment have identified dosing errors in up to one-third of antimicrobial prescriptions [23]. Similar findings have been reported in both tertiary-care hospitals and community healthcare settings, suggesting that this issue is not limited to a particular type of institution [34,35]. Differences in error rates may also reflect variations in the availability of clinical decision support tools, pharmacist involvement in antimicrobial stewardship programs, and institutional prescribing practices. However, the rate of inappropriately dosed antimicrobials in this study is very high and efforts to reduce inappropriate dosing should be made to increase patient safety by optimizing antimicrobial activity and reducing the possibility of adverse events and drug–drug interactions.

Patients receiving incorrect antimicrobial doses were significantly older and had worse renal function, including lower GFR, and higher rates of dialysis dependence. These findings are consistent with the literature, which reports that advanced age, chronic kidney disease, and acute kidney injury are among the strongest predictors of antimicrobial dosing errors [31,32,36]. Age-related physiological changes, multimorbidity, and fluctuating renal function likely contribute to the complexity of dose adjustment in elderly hospitalized patients, increasing the risk of both overdosing and underdosing [8]. Interestingly, in a multivariate logistic regression analysis evaluating all parameters that were found to differ in a statistically significant manner between appropriately and inappropriately dosed antimicrobials, only GFR was identified as an independent predictor of inappropriate dosing of antimicrobials, with low GFR increasing the likelihood of dose inappropriateness. More specifically, for each added mL/min/1.73 m^2^, there was an odds ratio of 0.979 for inappropriate dose of antimicrobials. This was largely anticipated, as inappropriate dosing of antimicrobials was defined based on the GFR. However, the value of this multivariate analysis is largely due to the exclusion of other factors as being independently associated with inappropriately dosed antimicrobials.

The predominance of overdosing observed in the present study is particularly concerning from a clinical safety perspective. Excessive antimicrobial exposure in patients with impaired renal function may lead to drug accumulation and increased risk of adverse effects [37]. Several antimicrobial classes are associated with well-recognized toxicity when administered at inappropriate doses in the setting of renal dysfunction. For instance, accumulation of β-lactam antibiotics has been linked to neurotoxicity manifesting as encephalopathy, confusion, myoclonus, or seizures, particularly in elderly patients or those with severe renal impairment [38]. Similarly, colistin is known for its nephrotoxic potential, and acyclovir may cause both nephrotoxicity and neurotoxicity when high plasma concentrations occur due to inadequate dose adjustment [39,40,41]. These adverse effects may prolong hospitalization, increase healthcare costs, and negatively affect patient outcomes.

Although less frequent than overdosing in this study, underdosing also represents an important clinical concern. Inadequate antimicrobial exposure may lead to suboptimal therapeutic outcomes, including delayed resolution of infection or treatment failure. Furthermore, exposure to subtherapeutic antimicrobial concentrations has been associated with the selection and propagation of antimicrobial-resistant organisms. This issue is particularly relevant for β-lactam antibiotics, whose pharmacodynamic efficacy depends on maintaining drug concentrations above the minimum inhibitory concentration for an adequate proportion of the dosing interval. In critically ill patients or those with severe infections, inappropriate dose reduction may therefore compromise the effectiveness of therapy [42].

Drug-specific analysis revealed substantial heterogeneity in dosing appropriateness. Piperacillin/tazobactam, the most commonly prescribed antimicrobial in the present study, showed a notable rate of underdosing, consistent with the literature, which recognizes it as a frequent source of renal dosing errors [31,43]. Given its widespread use and time-dependent pharmacodynamics, inappropriate dose reduction may compromise clinical efficacy. High error rates observed with ceftazidime, cefuroxime, colistin, and acyclovir likely reflect lower prescriber familiarity with renal dosing recommendations and the complexity of dose adjustment for these agents, particularly in patients with advanced kidney disease or those receiving dialysis [8]. More specifically, colistin, which is considered a last-resort antimicrobial but is used very often in Greece due to the frequency of infections by extensively drug-resistant bacteria such as *Acinetobacter baumannii*, may be prescribed incorrectly due to its complexity that is highly dependent on the patient’s GFR [44,45,46,47]. On the other hand, acyclovir, a less frequently prescribed antiviral, also has high dependency on the patient’s GFR that could lead to prescription errors [48]. Similarly, cefuroxime, a cephalosporin that has a different dosing pattern intravenously than its oral counterpart, and is less frequently prescribed compared to other cephalosporins, such as ceftriaxone, could also be prescribed incorrectly in patients with abnormal GFR [49].

The association between dosing inappropriateness and the number of concurrently prescribed antimicrobials suggests that polypharmacy further increases the risk of prescribing errors. Similar associations have been reported in other studies [50]. Clinical pharmacist involvement, automated renal dosing alerts, and standardized dosing protocols have been shown to improve dosing accuracy and reduce preventable adverse drug events [51,52,53].

The findings of the present study highlight the importance of antimicrobial stewardship programs in optimizing antimicrobial dosing practices. Stewardship initiatives focusing on renal dose adjustment may include educational interventions for prescribers, integration of dosing guidelines into electronic prescribing systems, and active involvement of clinical pharmacists in medication review [54,55]. Clinical decision support systems integrated within electronic health records have been shown to reduce dosing errors by providing automated alerts or dosing recommendations based on renal function parameters [56,57]. Additionally, pharmacist-led antimicrobial stewardship interventions have demonstrated significant improvements in dosing accuracy and reductions in adverse drug events [58,59]. To that end, implementation of such practices, such as unsolicited consultations by infectious diseases physicians, educational interventions to prescribers of antimicrobials, and possibly, clinical decision support systems integrated within electronic health records could be implemented in the present hospital to reduce dosing errors in antimicrobial prescribing. On the other hand, local prescribing practices and different antimicrobial stewardship infrastructures in other hospital settings can limit generalizability of the results of the present study. Thus, physicians should take into account important differences in antimicrobial prescribing practices when taking into consideration the results of the present study.

Another important consideration in antimicrobial dosing is the dynamic nature of renal function in hospitalized patients. Acute kidney injury may develop rapidly during hospitalization due to infection-related hemodynamic instability, nephrotoxic medications, or underlying comorbidities [60]. Conversely, renal function may improve following appropriate treatment and supportive care. Therefore, antimicrobial dosing should not be considered a static decision but rather a process requiring ongoing reassessment. Regular monitoring of renal function and timely adjustment of antimicrobial therapy are essential to maintain appropriate drug exposure throughout the course of treatment.

Several limitations should be considered when interpreting the results of this study. First, the point-prevalence design provides a cross-sectional snapshot of antimicrobial prescribing practices relying on a single day and does not capture changes in dosing that may occur during the course of hospitalization. As renal function can fluctuate rapidly and significantly in hospitalized patients, particularly those with acute illness, some prescriptions classified as inappropriate at the time of the survey may have been subsequently adjusted. Secondly, the study was conducted at a single tertiary-care hospital, which may limit the generalizability of the findings to other healthcare settings. Prescribing practices, availability of antimicrobial stewardship resources, and patient populations may differ across institutions and healthcare systems. On the other hand, the number of patients in the ICU in this study was small, given that the hospital has about 20 ICU beds; thus, the results of the present study may not be representative of the prescribing behavior in critically ill patients. Thirdly, the study did not evaluate clinical information such as body weight, height, or body mass index, comorbidities, or patients’ clinical outcomes associated with dosing inappropriateness, such as treatment failure, adverse drug reactions, or mortality. Assessing the clinical impact of dosing errors would provide valuable insight into the real-world consequences of inappropriate antimicrobial dosing. Additionally, the GFR was estimated using the MDRD equation since this is easily estimated via the electronic health system used for patients’ records and does not require patients’ weight, which is not readily available from all patients, nor is it always accurate in all clinical scenarios. However, most antimicrobial dosing recommendations are based on creatinine clearance calculated using the Cockcroft–Gault formula. The use of MDRD could have misclassified renal function categories and dosing appropriateness in some instances. Moreover, this study did not make any distinction on whether the low GFR was due to acute kidney injury or chronic kidney failure, given that antimicrobials are dosed based on the GFR based on the currently available protocols, without any differences if the low GFR is attributable to acute kidney injury or chronic kidney disease. However, distinguishing between acute kidney injury and chronic kidney disease could have provided important information since these two conditions differ significantly in terms of pharmacokinetics. Finally, as this study was a PPS, we chose to perform it on a single day in the middle of the working week, away from public or local holidays, so that it could be as representative as possible. However, the possibility of it not being fully representative of regular hospital practices may, theoretically, still exist.

Future research should aim to evaluate the effectiveness of targeted stewardship interventions designed to improve renal dose adjustment practices. Prospective studies incorporating automated clinical decision support tools, pharmacist-led interventions, or educational programs may help identify strategies that effectively reduce dosing errors and improve patient outcomes. Finally, the GFR was calculated using the MDRD formula, not the Cockcroft–Gault formula, due to the data provided from the hospital’s electronic system.

## 5. Conclusions

In conclusion, inappropriate antimicrobial dosing in relation to kidney function remains common in hospitalized patients, particularly among the elderly, those with impaired renal function, and those receiving multiple antimicrobials. Focused antimicrobial stewardship interventions emphasizing renal dose adjustment, especially for drugs found to be prescribed incorrectly often and high-risk patient populations, are warranted to improve the safety and effectiveness of antimicrobial therapy.

## Figures and Tables

**Table 1:** Patients’ characteristics overall and whether antimicrobial dosing was correct.

Characteristic	All Patients (*n* = 235)	Correct Dose (*n* = 209)	Wrong Dose (*n* = 37)	*p*-Value
Age, years, mean, (SD)	64.8 (18.6)	64.1 (18.7)	71.5 (15)	0.0220
Female gender, *n* (%)	102 (43.4)	95 (45.5)	12 (32.4)	0.1541
Ward type				
Medical, *n* (%)	121 (51.5)	110 (52.6)	19 (51.4)	1
Surgical, *n* (%)	108 (46)	95 (45.5)	16 (43.2)	0.8590
ICU, *n* (%)	6 (2.6)	4 (1.9)	2 (5.4)	0.2204
Serum creatinine, mg/dL, mean (SD)	1.27 (1.2)	1.19 (1.2)	1.68 (1.3)	0.0174
GFR, mL/min/1.73 m^2^, mean (SD)	73.1 (29.4)	75.9 (28.2)	58.5 (31.6)	0.0009
Patient on dialysis, *n* (%)	12 (5.1)	9 (4.3)	5 (13.5)	0.0422
Number of antibiotics prescribed, median (IQR)	1 (1–2)	1 (1–2)	2 (1–3)	0.0185
Way of administration				
Intravenous, *n* (%)	348 (87.8)	315 (88.2)	33 (84.6)	0.4485
Orally, *n* (%)	46 (11.6)	40 (11.2)	6 (15.4)	0.4308
Most common antimicrobials				
Piperacillin/tazobactam, *n* (%)	50 (12.6)	43 (12)	7 (17.9)	
Cefoxitin, *n* (%)	35 (8.8)	31 (8.7)	4 (10.3)	
Ceftriaxone, *n* (%)	28 (7.1)	28 (7.8)	0 (0)	
Metronidazole, *n* (%)	28 (7.1)	27 (7.6)	1 (2.3)	
Meropenem, *n* (%)	25 (6.3)	23 (6.4)	2 (5.1)	

GFR: glomerular filtration rate; ICU: intensive care unit; IQR: interquartile range; SD: standard deviation.

**Table 2:** Patients’ characteristics in terms of whether antimicrobial dosing was over or under the indicated, based on patient’s kidney function.

Characteristic	Overdosing (*n* = 23)	Underdosing (*n* = 16)	*p*-Value
Age, years, mean, (SD)	75.6 (10.8)	65.8 (17.6)	0.0369
Female gender, *n* (%)	9 (39.1)	3 (18.8)	0.2913
Ward type			
Medical, *n* (%)	15 (65.2)	4 (25)	0.0225
Surgical, *n* (%)	8 (34.8)	10 (62.5)	0.1122
ICU, *n* (%)	0 (0)	2 (12.5)	0.1619
Serum creatinine, mg/dL, mean (SD)	1.91 (1.4)	1.26 (0.9)	0.1076
GFR, mL/min/1.73 m^2^, mean (SD)	49 (29.3)	74.1 (27.8)	0.0108
Patient on dialysis, *n* (%)	4 (17.4)	1 (6.3)	0.3848
Number of antibiotics prescribed, median (IQR)	3 (1–3)	1 (1–2)	0.0522
Way of administration			
Intravenous, *n* (%)	19 (82.6)	14 (87.5)	1
Orally, *n* (%)	4 (17.4)	2 (12.5)	1
Most common antimicrobials			
Piperacillin/tazobactam, *n* (%)	2 (8.7)	5 (31.3)	
Cefoxitin, *n* (%)	1 (4.3)	3 (18.8)	
Ciprofloxacin, *n* (%)	1 (4.3)	1 (6.3)	
Meropenem, *n* (%)	1 (4.3)	1 (6.3)	
Ceftazidime, *n* (%)	2 (8.7)	0 (0)	

GFR: glomerular filtration rate; ICU: intensive care unit; IQR: interquartile range; SD: standard deviation.

**Table 3:** Results of the multivariate logistic regression analysis of inappropriate dosing of antimicrobials.

Characteristic	Multivariate Analysis *p*-Value	OR (95% CI)
Age, per year	0.351	1.012 (0.987–1.038)
Creatinine, per mg/dL	0.267	0.759 (0.466–1.235)
GFR, per mL/min/1.73 m^2^	0.030	0.979 (0.961–0.998)
Number of antimicrobials used, per each one	0.856	1.028 (0.762–1.386)

CI: confidence interval; OR: odds ratio.

**Table 4:** Data of most commonly prescribed antimicrobials with incorrect dosing.

Antimicrobial	Number of Prescriptions (*n*)	Percent of Wrong Prescriptions	Percent of Prescriptions with Higher Dose	Percent of Prescriptions with a Lower Dose
Piperacillin/tazobactam	50	14	4	10
Cefoxitin	35	11.4	2.9	8.6
Meropenem	25	8	4	4
Ciprofloxacin	11	18.2	9.1	9.1
Colistin	6	33.3	16.7	16.7
Ceftazidime	5	40	40	0
Cefuroxime	5	40	0	40
Acyclovir	3	33.3	33.3	0

## Data Availability

Data are available from the authors upon reasonable request.
